# Perforated Gastric Cancer: Epidemiology, Diagnosis, and Surgical Management Strategies

**DOI:** 10.3390/jcm15155931

**Published:** 2026-07-29

**Authors:** Sang-Ho Jeong, Sung Jin Oh, Kyung Won Seo, Jae-Seok Min

**Affiliations:** 1 Department of Surgery, Gyeongsang National University College of Medicine and Gyeongsang National University Changwon Hospital, Changwon 51472, Republic of Korea; shjeong@gnu.ac.kr; 2 Department of Surgery, Haeundae Paik Hospital, Inje University College of Medicine, Busan 48108, Republic of Korea; 3 Department of Surgery, Kosin University Gospel Hospital, Busan 49267, Republic of Korea; 4 Division of Foregut Surgery, Department of Surgery, Korea University College of Medicine, Korea University Anam Hospital, Seoul 02841, Republic of Korea

**Keywords:** gastric neoplasm, perforation, peritonitis, emergency surgery, gastrectomy, two-stage surgery, R0 resection

## Abstract

Gastric cancer complicated by free perforation is a rare but life-threatening oncological emergency, accounting for 0.3% ~ 3.9% of all gastric cancer cases. This review summarizes current evidence on the epidemiology, diagnosis, surgical management, and prognostic determinants of perforated gastric cancer, with emphasis on stage-adapted treatment strategies. It should be noted, however, that the available evidence is derived exclusively from retrospective studies, and all recommendations should be interpreted in the context of this inherent limitation. Relevant studies addressing perforation patterns, perioperative outcomes, one-stage versus two-stage gastrectomy, repair-only approaches, and management of metastatic disease were reviewed. Perforation typically arises from tumors located on the anterior wall or greater curvature, where transmural invasion creates direct communication with the peritoneal cavity, resulting in diffuse peritonitis and substantial postoperative mortality. Available pooled evidence suggests that radical resection, when oncologically and physiologically feasible, is associated with better survival than repair-only strategies. Comparative retrospective data suggest that two-stage gastrectomy may be associated with higher R0 resection rates and lower in-hospital mortality compared with emergency one-stage resection, without compromising long-term survival when curative resection is ultimately achieved; however, these findings should be interpreted with caution given the exclusively retrospective evidence base and the inherent patient selection bias in all published comparisons. In patients with stage IV disease or confirmed distant metastases, stomach-preserving source control followed by systemic chemotherapy may be a rational alternative. Prognosis is primarily determined by TNM stage and R0 resection status, similar to non-perforated gastric cancer. Individualized treatment based on tumor stage, resectability, peritoneal contamination, and physiological reserve is essential.

## 1. Introduction

Gastric cancer remains one of the leading causes of cancer-related mortality worldwide, accounting for approximately 770,000 deaths annually [1]. Its clinical course may be complicated by life-threatening local emergencies, of which massive hemorrhage and free perforation are the most consequential [2,3]. While hemorrhage occurs more commonly and typically originates from tumors involving the lesser curvature or posterior gastric wall—regions abundantly supplied by perigastric vessels—perforation arises predominantly from tumors on the anterior wall or greater curvature, where progressive transmural invasion leads to free communication with the peritoneal cavity [1,2]. This anatomical distinction has direct implications for both the mode of clinical presentation and the appropriate surgical response.

Free perforation of a gastric cancer results in spillage of intragastric contents and tumor cells into the peritoneal cavity, triggering generalized peritonitis analogous to that caused by benign peptic ulcer perforation. However, the clinical context is substantially more demanding: affected patients are typically elderly, nutritionally compromised, and harbor advanced-stage disease at the time of the event. Free perforation complicates fewer than 3% of all gastric cancers, yet it carries disproportionately high perioperative morbidity and mortality, with postoperative death rates reported between 10% and 40% across published series [1,2,3,4].

A distinctive challenge of perforated gastric cancer lies in the frequent absence of a preoperative tissue diagnosis. Emergency laparotomy for acute peritonitis is typically performed without prior upper endoscopy or biopsy, and intraoperative frozen-section analysis—though desirable—is rarely feasible outside regular working hours. Surgeons must therefore make consequential operative decisions under conditions of diagnostic uncertainty, physiological instability, and time pressure [2,5,6].

Despite its rarity, perforated gastric cancer has attracted increasing research attention as evidence has accumulated demonstrating that the surgical strategy—ranging from simple closure alone to immediate radical gastrectomy, and from single-stage to two-stage resection—substantially influences both short-term survival and long-term oncological outcomes. The present review synthesizes current evidence on the epidemiology, clinical presentation, computed tomographic diagnosis, and surgical management of perforated gastric cancer, with the aim of supporting individualized, evidence-based decision-making in this challenging emergency context.

## 2. Literature Search Strategy

This narrative review was conducted through a systematic search of the PubMed/MEDLINE, Embase, and Cochrane Library databases, covering publications from January 1990 to March 2026. The following Medical Subject Headings (MeSH) terms and free-text keywords were used in various combinations: “stomach neoplasms,” “gastric cancer,” “perforation,” “perforated gastric cancer,” “gastrectomy,” “damage control surgery,” “two-stage gastrectomy,” “peritoneal lavage cytology,” “ramucirumab,” “anti-angiogenic therapy,” and “prognosis.” Searches were limited to publications in the English language. Studies were considered eligible for inclusion if they reported original clinical data on patients with perforated gastric cancer, including randomized controlled trials, prospective or retrospective cohort studies, and case series of five or more patients. Systematic reviews and meta-analyses addressing surgical outcomes, prognostic factors, or chemotherapy-related perforation were also included. Single case reports were excluded from the main synthesis but were selectively referenced in the section on chemotherapy-associated perforation, given the rarity of this clinical entity and the limited available evidence. This review did not follow a pre-registered protocol and was not designed as a systematic review; accordingly, PRISMA guidelines were not applied. The selection of studies and topics was guided by clinical relevance and the aim of providing a comprehensive, evidence-based framework for the surgical management of this rare oncological emergency.

## 3. Epidemiology and Risk Factors

Perforation complicates fewer than 3% of all gastric cancer cases, with reported incidence ranging from 0.3% to 3.9% across published series [2,3,4,7]. It constitutes the second most frequent oncological emergency in gastric cancer after severe hemorrhage [8]. A 2021 systematic appraisal pooling 628 patients from 11 studies demonstrated that perforation predominates among patients with advanced-stage disease, with the majority harboring stage III or IV tumors at the time of the acute event [4]. Nevertheless, perforation can occur in early gastric cancer, a finding with important surgical implications as it expands the potential eligibility for curative resection [9,10,11].

The epidemiological profile of perforated gastric cancer displays a characteristic predilection for older male patients. The majority of affected individuals are men in their sixth decade of life, and the incidence appears higher in Western than Asian populations—possibly reflecting regional differences in tumor histotype distribution, anatomical tumor location, and access to early detection programs [2,12]. In the emergency setting, this demographic pattern constitutes an important diagnostic cue: among patients presenting with gastroduodenal perforation and peritonitis who are 60 years of age or older, the possibility of an underlying malignancy must be explicitly considered and pursued even when preoperative endoscopic confirmation is unavailable [1,2].

Among risk factors associated with perforation specifically, tumor location on the anterior gastric wall or greater curvature is the most consistently reported anatomical predisposing feature [1,2]. The use of anti-angiogenic agents—most notably ramucirumab, a VEGFR-2 antagonist employed in second-line therapy for metastatic gastric cancer—represents a growing iatrogenic category of perforation risk, through mechanisms involving impaired vascular repair, tumor ischemia, and transmural necrosis [13,14]. Preoperative exposure to non-steroidal anti-inflammatory drugs (NSAIDs) and corticosteroids, chronic Helicobacter pylori-associated mucosal damage underlying the tumor, and tumor necrosis in the setting of locally advanced disease have also been implicated as contributing factors.

## 4. Clinical Presentation and Diagnosis

Patients with perforated gastric cancer typically present with the sudden onset of severe, diffuse abdominal pain and the clinical signs of peritonitis—including involuntary guarding, board-like rigidity, and rebound tenderness—accompanied by systemic inflammatory response features such as tachycardia, fever, and, in advanced cases, frank hemodynamic compromise. This presentation is clinically indistinguishable from that of benign peptic ulcer perforation, and a correct preoperative diagnosis of an underlying malignancy is established in only approximately one-third of cases [8].

**Figure 1:**
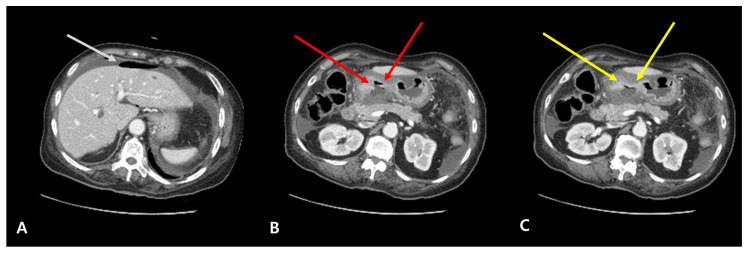
Abdominal computed tomography (CT) scans showing gastric cancer perforation. (**A**) Pneumoperitoneum with free intraperitoneal air (white arrow) is noted in the upper abdomen. (**B**) The perforation site is identified by a localized defect in the gastric wall (red arrows). (**C**) Marked, irregular thickening of the gastric wall (yellow arrows) is well-visualized, highly suggestive of the underlying gastric malignancy.

Initial evaluation includes erect chest radiography, which may reveal pneumoperitoneum as a subphrenic lucency, and contrast-enhanced computed tomography (CT) of the abdomen and pelvis, which has become the cornerstone of emergency imaging in this setting. CT allows the detection of free intraperitoneal air, free fluid, focal gastric wall thickening or discontinuity, perigastric fat stranding, and—when present—regional lymphadenopathy or distant metastatic deposits. In the upper abdomen, pneumoperitoneum with free intraperitoneal air was prominently noted (Figure 1A, white arrow). The exact perforation site was identified by a localized defect along the gastric wall (Figure 1B, red arrows). Furthermore, marked and irregular thickening of the gastric wall was well-visualized (Figure 1C, yellow arrows), which was highly suggestive of the underlying gastric malignancy. In terms of diagnostic performance, multidetector CT (MDCT) achieves near-100% sensitivity for the detection of pneumoperitoneum, and when wall defect and wall thickening are evaluated in combination, perforation site localization reaches 95% sensitivity and 93% specificity [15]. Several CT features may assist in distinguishing a malignant from a benign etiology of gastric perforation. Features more suggestive of an underlying malignancy include nodular or irregular wall thickening with soft-tissue rather than edematous low-attenuation enhancement, the presence of perigastric or regional lymphadenopathy, distant metastatic deposits, and perforation arising within or adjacent to an ulcerated mass rather than a clean-edged mucosal defect [16,17]. In contrast, benign peptic ulcer perforation more commonly presents with smooth, symmetric wall thickening, edematous low-attenuation enhancement, and the absence of lymphadenopathy or metastatic disease. However, these features overlap substantially in the acute setting, and CT cannot reliably exclude malignancy in a significant proportion of cases [18].

Several important diagnostic pitfalls merit recognition. First, in covered or contained perforations—where the defect is sealed by adherent omentum or adjacent organs—free intraperitoneal air may be absent or minimal, substantially reducing CT sensitivity and potentially delaying the diagnosis of perforation altogether. Second, emergency CT is typically performed without oral contrast and under conditions of gastric underdistension, which may limit visualization of intraluminal pathology and lead to underestimation of wall abnormalities. Third, diffuse-type gastric adenocarcinoma (signet-ring cell or poorly cohesive carcinoma) may infiltrate the gastric wall without forming a discrete mass lesion, rendering its CT appearance particularly subtle and easily mistaken for benign inflammatory thickening [18]. These limitations underscore the importance of maintaining a high clinical index of suspicion for malignancy based on patient demographics and clinical history, even when CT findings appear non-specific, and of submitting all perforation-edge biopsy material for definitive histopathological examination. Although CT findings are frequently non-specific in the acute phase, a combination of these features—particularly irregular or indurated gastric wall thickening rather than a smooth-edged ulcer crater—may heighten suspicion for malignancy and guide intraoperative decision-making [19].

A key epidemiological diagnostic clue in the emergency setting is patient age. Because perforated gastric cancer occurs in a substantially older population than peptic ulcer perforation, clinical suspicion for malignancy should be heightened in patients over 60 years presenting with gastroduodenal perforation [1,2]. Additional clinical features that may raise suspicion include a prolonged antecedent history of dyspeptic symptoms, significant unintentional weight loss, iron-deficiency anemia, and the absence of conventional ulcer risk factors such as known NSAID use or prior peptic ulcer disease.

**Figure 2:**
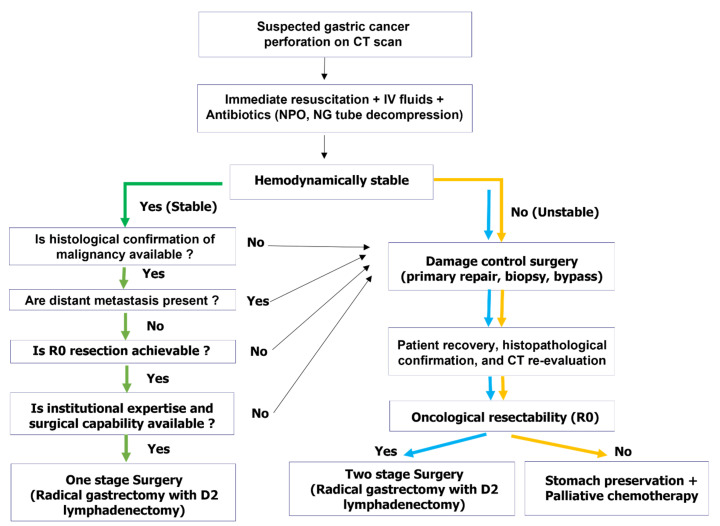
Surgical decision algorithm for perforated gastric cancer. All patients initially receive resuscitation (IV fluids, broad-spectrum antibiotics, nil by mouth, nasogastric tube decompression), followed by hemodynamic assessment as the primary decision node. Hemodynamically stable patients (green pathway, left) proceed through four sequential binary assessments prior to definitive surgery: Step 1—Is histological confirmation of malignancy available? (No → damage-control surgery); Step 2—Are distant metastases present? (Yes → damage-control surgery and palliative management; No → proceed to Step 3); Step 3—Is R0 resection oncologically achievable? (No → damage-control surgery); Step 4—Is adequate surgical expertise and institutional capability available? (No → damage-control surgery with transfer to specialist center; Yes → immediate one-stage radical gastrectomy with D2 lymphadenectomy). When all four criteria are satisfied, immediate one-stage radical gastrectomy with D2 lymphadenectomy is performed. Hemodynamically unstable patients or those progressing to sepsis (yellow/blue pathway, right) proceed directly to damage-control surgery (primary repair, biopsy, or bypass), followed by patient recovery, histopathological confirmation, and CT re-evaluation. Oncological resectability is then reassessed: unresectable disease → stomach preservation and palliative chemotherapy (yellow pathway); resectable disease → planned two-stage radical gastrectomy (blue pathway). Color coding: green, stepwise evaluation leading to one-stage radical gastrectomy; yellow, stomach preservation and palliative chemotherapy; blue, two-stage radical gastrectomy.

Although upper gastrointestinal endoscopy with biopsy remains the diagnostic gold standard for gastric cancer, it is rarely feasible in the acute phase of peritonitis. Intraoperative frozen-section analysis of the perforation edge is the preferred approach when logistically available, as it enables real-time surgical planning. However, resource and timing constraints—particularly emergency operations outside working hours—limit its practical use [2,5,6]. Macroscopic features at laparotomy that should raise suspicion for malignancy include indurated, irregular perforation margins, abnormal mucosal texture, adherent omentum, and grossly enlarged regional lymph nodes. All resected or biopsied tissue from emergency gastric procedures must be submitted for definitive histopathological examination irrespective of the intraoperative impression. In hemodynamically stable patients with suspected perforated gastric cancer, emergency diagnostic laparoscopy offers a valuable adjunct to the diagnostic and staging workup prior to definitive surgery. Under direct laparoscopic visualization, surgeons can confirm the perforation site and assess its characteristics, collect peritoneal lavage fluid for cytological examination, obtain targeted biopsies of the perforation edge for intraoperative or permanent histopathological analysis, and perform initial peritoneal lavage as a component of damage control. These capabilities directly facilitate the individualized decision-making outlined in the surgical algorithm (Figure 2), enabling real-time assessment of resectability, peritoneal dissemination, and the feasibility of curative-intent resection before committing to a definitive operative strategy.

## 5. Surgical Management

### 5.1. Principles of Surgical Decision-Making

The surgical management of perforated gastric cancer requires individualization based on a comprehensive real-time assessment of five determinants: (1) availability of a preoperative or intraoperative histological diagnosis of malignancy; (2) the patient’s age and physiological reserve, encompassing hemodynamic stability, ASA classification, and the burden of comorbid disease; (3) the presence of overt peritoneal carcinomatosis or distant metastases identified at laparotomy; (4) perforation characteristics including anatomical location, size, degree of peritoneal contamination, and tissue quality at the perforation edge; and (5) the operating surgeon’s proficiency with emergency radical gastrectomy and intracorporeal reconstruction [2,5,6]. These factors must be rapidly integrated against the overarching oncological principle that R0 resection—when physiologically and anatomically achievable—confers the greatest long-term survival benefit.

Critically, histological confirmation of malignancy and assessment of oncological resectability constitute distinct and sequential decision points that must not be conflated: the absence of confirmed histology alone may be sufficient reason to defer radical resection to a planned second-stage procedure, even in patients who are otherwise hemodynamically stable. The surgical decision algorithm is presented in Figure 2, incorporating two primary pathways determined by hemodynamic status, each with explicit sequential assessment of histological confirmation, the presence of distant metastases, oncological resectability, and institutional surgical expertise. In hemodynamically stable patients, all four criteria must be satisfied before proceeding to immediate one-stage radical gastrectomy; failure to meet any single criterion redirects to the damage-control pathway. In hemodynamically unstable patients, damage-control surgery is performed as the first stage, with oncological resectability reassessed after recovery to determine between two-stage radical gastrectomy and stomach-preserving palliative management.

### 5.2. Simple Closure Versus Radical Resection

Historically, the perceived risks of radical emergency surgery in the setting of peritoneal contamination led to a preference for simple closure—primary suture repair with or without omental patch—as the definitive operative approach, particularly based on the assumption that intraperitoneal tumor cell spillage at the time of perforation rendered curative intent futile [3,20,21,22]. This conservative paradigm has been progressively challenged by accumulating evidence demonstrating that the surgical principles governing non-perforated gastric cancer apply equally to the perforated setting when patient physiology permits.

A 2021 critical appraisal of 628 pooled patients from 11 studies found that radical resection—whether one-stage or two-stage—was associated with significantly superior survival compared to repair-only surgery, with gastrectomy consistently associated with better long-term outcomes across subgroups [4]. The survival differential is most starkly illustrated by the comparison of median OS: R0 gastrectomy is associated with a median OS of approximately 21 months, versus only 4 months following simple closure or palliative resection [23]. When the patient is hemodynamically stable, peritoneal contamination is limited, and septic shock is absent, radical resection with D2 lymphadenectomy—executed according to established oncological standards—is the approach most likely to maximize survival [3,6,9,10,11].

The extent of lymphadenectomy in the emergency context remains debated. Perigastric edema and inflammatory change may complicate nodal dissection and impair accurate intraoperative staging. Nevertheless, available data consistently support the pursuit of D2 lymphadenectomy when anatomically feasible, as nodal harvest quality correlates directly with both staging accuracy and oncological outcome [8,23,24].

### 5.3. One-Stage Versus Two-Stage Gastrectomy

The central operative controversy in perforated gastric cancer management is the timing of radical resection: whether to perform definitive gastrectomy as a single emergency procedure or to adopt a damage-control strategy with delayed curative intent [23,25,26].

In one-stage gastrectomy, radical resection with anastomotic reconstruction is performed at the time of the emergency laparotomy. This eliminates the need for a second major procedure but demands high technical expertise in an uncontrolled operative environment, carries elevated anastomotic complication risk in the setting of inflamed and contaminated bowel, and forecloses the opportunity for preoperative oncological staging and planning.

Two-stage gastrectomy separates care into a damage-control phase—peritoneal lavage, simple closure or partial gastrectomy, and drain placement—followed, after physiological stabilization and definitive pathological staging, by planned radical gastrectomy with D2 lymphadenectomy [25]. This approach is particularly appropriate for patients presenting in shock, those operated outside regular hours when frozen-section analysis is unavailable, and cases in which perigastric inflammatory changes risk overestimating the extent of nodal disease. A recent single-institution 27-year retrospective series (1998–2023) confirmed the feasibility and safety of the two-stage approach, with all patients who underwent second-stage radical resection achieving curative-intent surgery without mortality [26].

The most comprehensive comparative evidence derives from a 2025 systematic review and meta-analysis by Manara et al. encompassing 13 studies and 621 patients, which reported no statistically significant difference in overall survival between one-stage and two-stage approaches [23]. However, two-stage gastrectomy achieved substantially higher R0 resection rates (78.4% vs. 50.0%) and markedly lower in-hospital mortality (1.9% vs. 11.4%). Individual patient data analysis further identified a trend toward higher mortality during the first 20 months of follow-up in the one-stage group, suggesting that two-stage resection may represent a lower-risk approach for patients with compromised physiology or in whom R0 resection cannot be confidently anticipated at the time of emergency laparotomy [23]. A critical interpretive caveat applies to all published comparisons between one-stage and two-stage gastrectomy: substantial patient selection bias is inherent in the study designs. Patients allocated to one-stage surgery are systematically more likely to be hemodynamically unstable, septic, and of advanced age, and to harbor more advanced disease with greater degrees of peritoneal contamination at the time of emergency laparotomy. These baseline differences independently predict worse short- and long-term outcomes, irrespective of operative timing or technique. Conversely, patients selected for two-stage surgery have typically survived the initial damage-control procedure, undergone physiological optimization, and completed definitive staging before radical resection—a process that inherently selects for a more favorable patient population. Although Zhang et al. employed propensity score matching in an attempt to control for measured confounders, unmeasured confounding by indication cannot be excluded in any of the available studies, and no randomized controlled trial exists to resolve this fundamental uncertainty [8,23]. The observed between-group differences in R0 resection rates and perioperative mortality should therefore be interpreted as reflecting a combination of operative strategy effects and patient selection effects, rather than attributable to surgical timing alone. These limitations must be explicitly acknowledged when translating the current evidence into clinical practice recommendations.

A further consideration limiting the generalizability of current evidence is the pronounced geographic and institutional concentration of available data. Virtually all landmark studies on perforated gastric cancer originate from high-volume tertiary centers in Japan, Korea, and China, where annual gastric cancer surgical volumes may exceed several hundred cases, dedicated gastric cancer surgical teams are available around the clock, intraoperative frozen-section pathology is routinely accessible, and proficiency in D2 lymphadenectomy is well established as an institutional standard [8,19,23,26]. These structural conditions differ fundamentally from those prevailing in Western hospitals, community emergency centers, and institutions without dedicated gastric cancer expertise, where perforated gastric cancer may be encountered only once or twice per decade, emergency radical gastrectomy may be performed by general surgeons without subspecialty training, and the infrastructure supporting complex intraoperative decision-making may be substantially more limited. In such settings, the R0 resection rates and perioperative outcomes reported from East Asian high-volume series are unlikely to be directly replicable, and a more conservative initial operative strategy—prioritizing safe source control and damage limitation over immediate radical resection—may represent the more prudent approach pending transfer to or consultation with a specialist center.

Hata et al. demonstrated that D2 resection was achieved in 72.0% of two-stage versus 28.7% of one-stage patients; crucially, survival after R0 resection did not differ significantly between the two strategies (*p* = 0.300), reaffirming that the achievement of R0 status—not the operative timing—is the dominant determinant of long-term prognosis [24]. Zhang et al., in a multicenter propensity score-matched analysis, further demonstrated significantly greater lymph node harvest in the two-stage group (31 vs. 17 nodes; *p* < 0.001) and longer median OS (45 vs. 11 months; *p* = 0.007), findings consistent with, though not conclusively attributable to, a higher oncological quality of resection in the two-stage cohort [8]. The primary disadvantage of the two-stage approach—the cumulative morbidity associated with two major operations—necessitates individualized patient-level judgment integrating institutional capabilities and operative expertise [25]. Taken together, these findings suggest that R0 resection status, rather than operative timing per se, may be the dominant determinant of long-term prognosis, and that the choice between one-stage and two-stage surgery must remain individualized as outlined above.

### 5.4. Stomach-Preserving Strategy for Stage IV Perforated Gastric Cancer

In patients with confirmed stage IV perforated gastric cancer and distant metastases, gastrectomy carries no curative intent and may prolong the recovery period, impair wound healing, delay chemotherapy initiation, and reduce overall quality of life. For such patients, a stomach-preserving strategy—comprising peritoneal lavage, source control via simple suture closure or omental patch repair, and early reinstitution of systemic chemotherapy—represents a rationally grounded clinical alternative [19].

Terayama et al. prospectively applied this approach in 10 patients with metastatic perforated gastric cancer, reporting zero cases of secondary perforation or anastomotic leak, oral intake resumption in 8 of 10 patients, and successful chemotherapy reinstitution in the majority. Median OS from surgery was 6 months, and median OS from initiation of chemotherapy was 12 months—outcomes consistent with stage IV gastric cancer managed in the non-emergency setting [19]. These data indicate that preserving the patient’s access to systemic therapy, rather than pursuing anatomical resection, should be the primary surgical objective in this population.

### 5.5. Chemotherapy-Associated Perforation

Gastric perforation may arise as a complication of systemic anticancer therapy, most notably with ramucirumab—a recombinant monoclonal antibody targeting VEGFR-2 employed as second-line treatment for advanced gastric and gastroesophageal junction adenocarcinoma. The mechanism involves VEGFR-2 blockade leading to impaired angiogenesis and vascular repair, tumor ischemia, compromised wound healing, transmural necrosis, and ultimately gastric or intestinal wall breakdown [13,14]. Importantly, ramucirumab-related perforation is not confined to the primary gastric tumor; perforation of small bowel metastatic deposits has been reported, underscoring the systemic nature of the risk [14].

Although definitive differentiation between drug-induced and spontaneous tumor perforation can be challenging, several clinical, radiological, and intraoperative features may assist in making this distinction. Clinically, drug-induced perforation typically occurs weeks to months after initiation of ramucirumab, with a reported all-grade incidence of 1.5% and a relative risk of 2.56 compared with controls in a pooled analysis of randomized controlled trials, and tends to arise at sites of tumor regression or necrosis rather than active invasion [27]. On contrast-enhanced CT, findings more suggestive of a drug-related etiology include intramural air (pneumatosis intestinalis), focal necrotic change within the tumor bulk, and a demonstrable reduction in tumor size compared with prior imaging; these contrast with the irregular wall thickening, surrounding lymphadenopathy, and serosal infiltration more characteristic of spontaneous perforation due to tumor progression [28]. Intraoperatively, drug-induced perforation tends to present with necrotic, relatively soft perforation margins and a less pronounced serosal inflammatory response, consistent with pathological findings of chemotherapy-induced tumor degeneration and necrosis at the perforation site rather than direct mucosal invasion [14]. Recognition of these distinguishing features is clinically important, as drug-induced perforation invariably occurs in the context of unresectable metastatic disease, where the primary surgical objective is restoration of gastrointestinal integrity to enable resumption of systemic therapy.

In patients receiving anti-angiogenic therapy who develop peritonitis, drug-associated perforation must be included in the differential diagnosis. Management in this setting should prioritize source control—simple suture closure or endoscopic ultrasound-guided drainage of intra-abdominal collections—with the therapeutic objective of enabling eventual resumption of chemotherapy rather than pursuing radical anatomical resection [13,14].

## 6. Prognosis and Prognostic Factors

The historically poor prognosis reported for perforated gastric cancer reflects the convergence of several adverse factors: most affected patients harbor advanced-stage disease, historical curative resection rates have been low owing to perceived contraindications of emergency surgery in a contaminated field, and the presumption of intraperitoneal tumor dissemination has historically led surgeons to forego radical resection [1,2,3]. Contemporary evidence has progressively revised this pessimistic view.

A fundamental insight from current data is that the prognostic determinants of perforated gastric cancer are identical to those of non-perforated disease. TNM stage—encompassing depth of tumor invasion (T), regional lymph node status (N), and distant metastasis (M)—remains the primary prognostic determinant [1,2,3,12]. In a pooled analysis of 628 cases, stage-stratified survival curves for perforated gastric cancer mirrored those reported for non-perforated disease, reinforcing that perforation per se is not an independent negative prognostic factor when disease stage and resection margin status are controlled [4]. Additional independent prognostic variables include serosal invasion, lymph node metastasis burden, the presence of distant metastasis, and—most critically—the achievement of R0 resection [23].

Peritoneal lavage cytology represents a specific prognostic tool in this context. Systematic reviews confirm that positive peritoneal cytology (CY1) is an independent predictor of recurrence and reduced survival in gastric cancer staged for curative resection [11]. Under both the JGCA 7th edition guidelines and the Korean Practice Guidelines for Gastric Cancer 2022, CY1 is classified as distant metastasis (M1) regardless of whether macroscopic peritoneal dissemination is present [29,30]. A clinically important distinction must be drawn between two scenarios: (1) when CY1 is identified preoperatively, its M1 classification should inform the decision against radical gastrectomy in favor of source control and systemic chemotherapy; (2) when CY1 is detected unexpectedly during emergency damage-control surgery, the JGCA guidelines suggest—with weak evidence (evidence level C) and as a conditional rather than general recommendation—that gastrectomy may be considered following multidisciplinary discussion and chemotherapy response assessment in selected patients [30]. The KGCA 2022 guidelines similarly classify CY1 as M1 and recommend systemic chemotherapy as the primary modality, with conversion surgery considered only in patients demonstrating a favorable response [29]. These guideline recommendations are derived from elective staging contexts, and their applicability to the acute gastric perforation setting remains uncertain.

The recognition that R0 resection—regardless of operative timing—appears to be the single most important modifiable survival determinant has substantially redirected contemporary management paradigms away from simple closure as definitive treatment and toward planned radical gastrectomy whenever patient condition and disease extent permit.

## 7. Future Perspectives

Several areas merit further investigation to optimize outcomes in perforated gastric cancer. As discussed in Section 3, diagnostic laparoscopy has already demonstrated utility in the pre-resection assessment of hemodynamically stable patients. The safety and oncological adequacy of minimally invasive emergency gastrectomy—laparoscopic or robotic-assisted—have been demonstrated in small case series and warrant formal prospective evaluation in experienced centers [26]. As laparoscopic proficiency in gastric surgery has expanded in East Asian high-volume institutions, the technical feasibility of laparoscopic omental patch repair as the damage-control first stage of a two-stage strategy is now well established; whether laparoscopic radical gastrectomy at either stage confers additional benefit in terms of reduced complication rates and recovery time remains to be defined in adequately powered studies.

The application of artificial intelligence (AI) to emergency CT interpretation represents a potentially transformative development for the early detection of malignancy in the acute setting. AI-based deep learning models capable of differentiating malignant from benign gastric wall pathology on non-contrast or contrast-enhanced CT have demonstrated promising diagnostic accuracy in preliminary studies [31], and their prospective validation in the emergency setting—where rapid triage of perforated gastric cancer from benign peptic ulcer perforation is clinically critical—would represent a meaningful advance. Integration of AI-assisted CT analysis into emergency imaging workflows could reduce diagnostic delays and support more timely, evidence-based operative decision-making.

Molecular biomarker testing at the time of emergency surgery represents another important emerging direction. Tissue obtained from perforation-edge biopsies or resected specimens during emergency gastrectomy is routinely submitted for histopathological examination, but is rarely assessed for actionable biomarkers such as HER2, PD-L1 combined positive score (CPS), microsatellite instability (MSI), and claudin 18.2 (CLDN18.2) expression—all of which now carry direct therapeutic implications in advanced gastric cancer [32]. Routine biomarker profiling of emergency specimens would enable oncological planning to commence immediately after histological confirmation, potentially accelerating the initiation of biomarker-directed systemic therapy in patients who have undergone damage-control surgery and are awaiting second-stage resection or systemic treatment.

Perioperative immunotherapy and conversion surgery represent an evolving frontier for patients with stage IV perforated gastric cancer. Immune checkpoint inhibitors—most notably nivolumab and pembrolizumab—have demonstrated activity in metastatic gastric cancer and are increasingly incorporated into first-line perioperative regimens [33]. In selected patients with perforated stage IV disease, a strategy of source control followed by immunotherapy-containing chemotherapy, with interval restaging and conversion surgery in patients achieving a significant response, merits prospective evaluation. Early case series have demonstrated the feasibility of conversion surgery following immunotherapy response in gastric cancer with peritoneal dissemination [34], and this paradigm could be extended to the post-perforation setting.

The role of hyperthermic intraperitoneal chemotherapy (HIPEC) at the time of emergency or elective second-stage gastrectomy in patients with limited peritoneal contamination or positive peritoneal cytology warrants investigation. A joint analysis of the European GASTRODATA Registry and the American National Cancer Database encompassing 193 patients with gastric cancer and peritoneal metastasis who underwent gastrectomy with HIPEC demonstrated feasibility across multinational settings [35]; whether these findings extend to the specific context of perforated gastric cancer, where peritoneal contamination is an additional variable, requires dedicated study. Ongoing randomized trials evaluating prophylactic HIPEC in high-risk gastric cancer (CHIMERA trial, NCT04597294) may generate evidence applicable to this population.

Finally, the development of international multicenter prospective registries represents the single most important infrastructural priority for advancing the evidence base in perforated gastric cancer. Given the rarity of the condition—affecting fewer than 3% of gastric cancer patients—no individual institution accumulates sufficient cases to power definitive comparative studies, and the entirety of available evidence derives from small retrospective series concentrated in East Asian high-volume centers. An international registry, incorporating standardized data collection on patient demographics, disease stage, operative strategy, perioperative management, biomarker profiles, and long-term oncological outcomes, would provide the foundation for hypothesis generation, subgroup analyses, and ultimately the design of pragmatic randomized trials. International surgical consortia have demonstrated the feasibility of such registries in gastric cancer surgery more broadly [36], and their extension to the specific context of perforated gastric cancer would represent a decisive step toward evidence-based management of this challenging emergency.

## 8. Limitations

Several important limitations of the existing evidence base deserve explicit acknowledgment. First, no randomized controlled trials have been conducted in the setting of perforated gastric cancer, and the entirety of comparative surgical evidence derives from retrospective, non-randomized studies of variable methodological quality. The absence of prospective data means that confounding by indication cannot be excluded, and causal inferences regarding the superiority of any particular surgical strategy must be drawn with circumspection. Second, patient selection bias is a pervasive limitation in all comparative studies of one-stage versus two-stage gastrectomy. Patients undergoing one-stage resection are disproportionately hemodynamically unstable, septic, older, and more likely to harbor advanced disease at the time of emergency laparotomy—characteristics that independently worsen surgical outcomes irrespective of the operative strategy chosen. This structural imbalance renders the reported differences in R0 resection rates and in-hospital mortality difficult to attribute unambiguously to operative timing alone. Third, the majority of available data originate from high-volume gastric cancer centers in Japan, Korea, and China. The structural differences between these institutions and Western or low-volume emergency centers are substantial and clinically consequential: East Asian high-volume centers typically maintain dedicated gastric cancer surgical teams with immediate availability, routine access to intraoperative frozen-section pathology, established D2 lymphadenectomy expertise, and high annual case volumes that collectively support complex emergency decision-making. These conditions cannot be assumed to exist in Western hospitals, community emergency departments, or institutions where perforated gastric cancer is encountered only sporadically. In such settings, attempting immediate radical gastrectomy in the absence of appropriate surgical expertise and infrastructure may expose patients to greater harm than a temporizing damage-control approach followed by transfer to a specialist center. The generalizability of current evidence-based recommendations to non-specialist settings therefore remains uncertain, and this limitation should be explicitly communicated to clinicians practicing outside high-volume gastric cancer centers. Fourth, individual study sample sizes are small—most published series include fewer than 50 patients with perforated gastric cancer—limiting the statistical power to detect clinically meaningful differences in survival outcomes and precluding robust subgroup analyses by disease stage, tumor location, or degree of peritoneal contamination. These limitations collectively underscore the need for multicenter prospective registries and, ultimately, pragmatic international trials to establish a higher-quality evidence base for the management of this rare oncological emergency.

## 9. Conclusions

Perforated gastric cancer is a rare but surgically demanding emergency requiring rapid, individualized decision-making across the dimensions of source control, hemodynamic resuscitation, operative extent, and oncological planning. The overarching principle governing contemporary management is that R0 resection—when physiologically and oncologically achievable—is the most important modifiable determinant of long-term survival, a conclusion congruent with the management of non-perforated disease.

Available retrospective data, predominantly from high-volume East Asian centers, suggest that a two-stage radical gastrectomy strategy may be associated with higher R0 resection rates and lower perioperative mortality compared with emergency one-stage resection in patients who are hemodynamically unstable, lack intraoperative histological confirmation, or cannot safely undergo immediate radical resection; long-term oncological outcomes appear comparable when curative resection is ultimately achieved. Given the exclusively retrospective nature of the available evidence, the inherent patient selection bias in all published comparisons, and the absence of randomized controlled trials, these observations should be interpreted as hypothesis-generating rather than practice-defining. Two-stage gastrectomy represents a reasonable approach to consider in appropriately selected patients at experienced institutions, but cannot be regarded as a universal standard of care. In patients with stage IV disease, stomach-preserving source control followed by systemic chemotherapy represents a feasible and rational alternative. Drug-associated perforation—particularly with ramucirumab—constitutes a distinct clinical entity requiring source-control-first management aimed at preserving access to anticancer therapy.

Future advances in this field will require prospective multicenter collaboration to standardize patient selection, evaluate minimally invasive emergency surgical approaches, and define the role of novel adjunctive strategies in a population where the urgency of presentation precludes conventional randomized trial design.

## Data Availability

No new data were created or analyzed in this study. Data sharing is not applicable to this article.
